# Whole Genome Sequencing of the Asian Arowana (*Scleropages formosus*) Provides Insights into the Evolution of Ray-Finned Fishes

**DOI:** 10.1093/gbe/evv186

**Published:** 2015-10-06

**Authors:** Christopher M. Austin, Mun Hua Tan, Larry J. Croft, Michael P. Hammer, Han Ming Gan

**Affiliations:** ^1^School of Science, Monash University Malaysia, Petaling Jaya, Selangor, Malaysia; ^2^Monash University Malaysia Genomics Facility, Monash University Malaysia, Petaling Jaya, Selangor, Malaysia; ^3^Malaysian Genomics Resource Centre Berhad, Boulevard Signature Office, Kuala Lumpur, Malaysia; ^4^Museum and Art Gallery of the Northern Territory, Darwin, NT, Australia

**Keywords:** genome, fish, phylogenomics, evolutionary rate, pigmentation genes

## Abstract

The Asian arowana (*Scleropages formosus*) is of commercial importance, conservation concern, and is a representative of one of the oldest lineages of ray-finned fish, the Osteoglossomorpha. To add to genomic knowledge of this species and the evolution of teleosts, the genome of a Malaysian specimen of arowana was sequenced. A draft genome is presented consisting of 42,110 scaffolds with a total size of 708 Mb (2.85% gaps) representing 93.95% of core eukaryotic genes. Using a k-mer-based method, a genome size of 900 Mb was also estimated. We present an update on the phylogenomics of fishes based on a total of 27 species (23 fish species and 4 tetrapods) using 177 orthologous proteins (71,360 amino acid sites), which supports established relationships except that arowana is placed as the sister lineage to all teleost clades (Bayesian posterior probability 1.00, bootstrap replicate 93%), that evolved after the teleost genome duplication event rather than the eels (Elopomorpha). Evolutionary rates are highly heterogeneous across the tree with fishes represented by both slowly and rapidly evolving lineages. A total of 94 putative pigment genes were identified, providing the impetus for development of molecular markers associated with the spectacular colored phenotypes found within this species.

## Introduction

More than half of all vertebrate species are fishes, with the Class Osteichthyes (bony fish) being the most diverse class within the Subphylum Vertebrata. (Santini et al. 2009; Near et al. 2012; Betancur-R et al. 2013). Fish have a long evolutionary history extending over 500 Myr into the Cambrian, with the evolution of the jawless fishes, which are currently represented by the lampreys (Agnatha). Jawed fishes (Gnathostoma) evolved some 450 Ma and are divided among three lineages: the cartilaginous fishes (Chondrichthyes), the bony fishes (Osteichthyes), and the lobe-finned fishes (Sarcopterygii). With the availability of more molecular genetic and genomic data, there has been increasing interest in understanding the diversification of the major fish groups and the molecular evolutionary dynamics of fish lineages, their timing, and evolution of specific genes (Inoue et al. 2003; Takezaki et al. 2004; Shan and Gras 2011; Near et al. 2012; Zou et al. 2012; Amemiya et al. 2013; Betancur-R et al. 2013; Broughton et al. 2013; Opazo et al. 2013; Dornburg et al. 2014; Venkatesh et al. 2014).

Of the 3 lineages in which fish are found, the bony fishes are by far the most diverse with nearly 30,000 recognized species and there has been much interest in understanding the drivers of their evolutionary success. Significant attention has been given to the impact of what is generally known as the fish- or teleost-specific genome duplication event (TGD) (Robinson-Rechavi et al. 2001; Hoegg et al. 2004; Hurley et al. 2005). Chromosomal duplications may provide opportunities for evolutionary experimentation, as paralogous genes are exapted to new functions, thereby facilitating rapid morphological, physiological, and behavioral diversification (Taylor et al. 2001; Hoegg et al. 2004; Meyer and Van de Peer 2005; Santini et al. 2009; Opazo et al. 2013).

The Asian arowana (*Scleropages formosus*: Osteoglossidae) is of fundamental interest to fish phylogenetics as it belongs to one of the oldest teleost groups, the Osteoglossomorpha. This lineage comprises the mooneyes, knifefish, elephantfish, freshwater butterflyfish, and bonytongues, and is one of the three ancient extant lineages that diverged immediately after the TGD. The other two are the Elopomorpha comprising eels, tarpons and bonefish, and the Clupeocephala, which embraces the majority of teleost diversity including the species-rich Ostariophysi (e.g., catfish, carps and minnows, tetras) and Percomorphaceae (e.g., wrasse, cichlids, gobies, flatfish) (Betancur-R et al. 2013; Broughton et al. 2013; Betancur-R, Naylor, et al. 2014; Betancur-R, Wiley, et al. 2014). There has been on-going disagreement on which one is the sister group to all other teleosts (Patterson and Rosen 1977; Nelson 1994; Arratia 1997; Patterson 1998; Zou et al. 2012). Historically, the Osteoglossomorph was considered to have diverged first (Patterson and Rosen 1977; Lauder and Liem 1983; Nelson 1994; Inoue et al. 2003; Brinkmann et al. 2004); however, comprehensive morphological studies, including both fossil and extant teleosts, and recent molecular-based studies supported the Elopomorpha as the sister lineage to all other bony fishes (Arratia 1997, 1999, 2000; Li and Wilson 1999; Diogo 2007; Santini et al. 2009; Near et al. 2012; Betancur-R et al. 2013; Broughton et al. 2013).

The arowana, sometimes also referred to as dragon fish, is also noteworthy as it is one of the most expensive fish in the world due to the occurrence of several bright color morphs that makes it highly sought after as an ornamental species (Dawes et al. 1999; Yue et al. 2006). Potentially relevant in this context is that teleost fishes are thought to have a greater range of pigment synthesis genes and pathways than any other vertebrate group (Braasch et al. 2009). However, the basis of color variation has seen little research in arowana with the exception of studies by Mohd-Shamsudin et al. (2011) and Mu et al. (2012) who found no consistent patterns of divergence between color variants and mitochondrial markers. *Scleropages formosus* is also of significant conservation concern in the wild. The species is listed by the International Union for Conservation of Nature (IUCN) as endangered (Kottelat 2013) and by the Convention on International Trades in Endangered Species of Wild Fauna and Flora as “highly endangered” (Yue et al. 2006).

In this study, we present the whole genome sequences for *S. formosus* obtained from a captive Malaysian specimen, as a representative of the local wild form. We then place this species within a phylogenetic framework including sequences from all available fish with sequenced genomes making this the most complete phylogenomic analysis of fish so far conducted. We also carry out analysis of the rate of molecular evolution within and between fish lineages and identify a range of genes associated with pigmentation.

### Genome Sequencing, Assembly, and Annotation

A total of 297,227,578 paired-end and 290,438,918 mate-pair reads (2 × 100 bp) were generated. Preprocessing resulted in 291,628,300 paired-end and 288,008,898 mate-pair reads, and these were subsequently assembled to generate a draft genome that consists of 42,110 scaffolds with a total size of 708 Mb and 2.85% gaps. The longest scaffold is 616,488 bp long and the N50 scaffold length is 58,849 bp. We also carried out a k-mer-based approach using read data and estimated the arowana genome size at approximately 900 Mb, a number in accord with the size of 1.05 Gb reported by Shen et al. (2014) estimated through flow cytometric comparative fluorescence with chicken cells. Based on these estimates, sequencing depth estimations ranging from 57 to 66 × coverage were inferred.

Features predicted from the assembly include 24,274 protein-coding genes, 609 transfer RNAs (tRNAs), and 29 ribosomal RNAs (100% 5S rRNA). Based on sequence similarity (*e*-value threshold of 1 × 10^−^^10^, hit coverage cut-off of 70%), 71% of the predicted genes shared sequence similarity to another protein in the nonredundant (NR) database on National Center for Biotechnology Information (NCBI). For protein-coding genes, 95.8% have Annotation Edit Distance (Eilbeck et al. 2009) scores of less than 0.5 and 85.5% contain at least one Pfam domain, an indication of a well-annotated genome (Campbell et al. 2014).

The gene space in this assembly appears fairly complete with 93.95% of core eukaryotic genes represented. This is further supported by the mapping of 78.92% of transcriptomic reads sequenced from a different arowana sample from Shen et al. (2014) to our assembled genome, with 64.32% of unmapped reads belonging to 18S and 28S ribosomal genes and 7.60% to mitochondrial genes. These genes are usually present in high copy numbers and may not have been assembled in our de novo assembly due to exceedingly high read coverage and short read lengths (Nagarajan and Pop 2013). This finding is also consistent with the lack of specific rRNAs (18S, 28S) predicted from the assembly.

### Phylogenomics and Evolutionary Rates

**Fig. 1.—:**
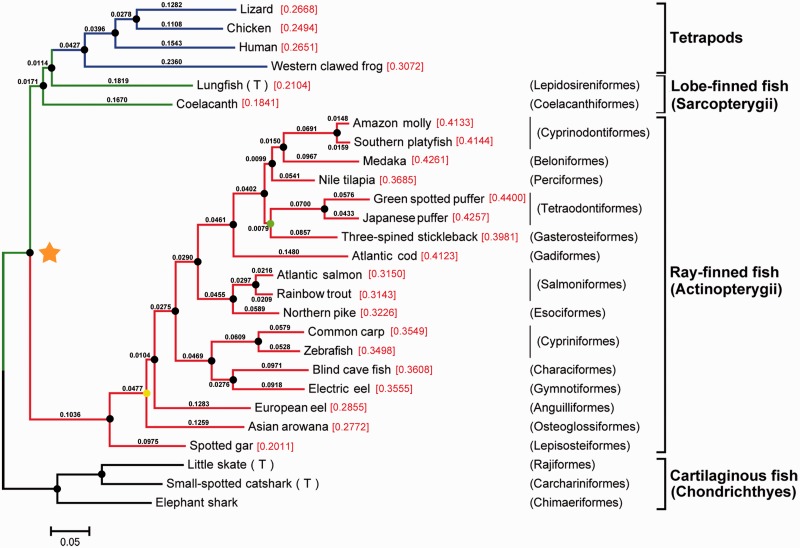
Phylogenetic relationships among fish species. The phylogenetic tree was inferred from a supermatrix containing the alignment of sequences from 27 species (177 orthologous proteins, 71,360 aligned amino acid positions, 7.07% gaps) and was rooted with the Chondrichthyes. Black circles indicate maximum nodal support with bootstrap values of 100% and Bayesian posterior probabilities of 1.00. The yellow and green circles represent 93% and 98% bootstrap support values, respectively, both with maximal Bayesian posterior probability values of 1.00. Branch length information is included and the rate of molecular evolution (number of amino acid substitutions per site) for each fish lineage is placed beside each taxa label. These values were calculated from the split of all ray-finned fish from lobe-finned fish and tetrapod lineages (node indicated with the orange star). A (T) is placed next to the species for which transcriptome data were utilized.

Our sample of arowana shows a 100% identity to the most common mitochondrial cytochrome c oxidase subunit 1 (COI) haplotype (accession number: HM156394) found among Malaysian specimens by Mohd-Shamsudin et al. (2011) and is 99.87% similar to the complete COI gene (accession number: DQ023143) from a fish obtained from a commercial farm in Singapore (Yue et al. 2006). Tree-based ortholog inference resulted in a set of orthologous proteins belonging to 177 gene families (supplementary material S1, Supplementary Material online) shared across all 23 fishes and 4 tetrapod species (table 1). Concatenation of each aligned ortholog generated a final supermatrix comprising of a total of 71,360 amino acid sites per species with only 7.07% gaps. The aligned supermatrix and the best-fit partitioning scheme generated by PartitionFinder can be found in supplementary materials S2 and S3, Supplementary Material online. Rooted with the Chondrichthyes, both Bayesian (BI) and maximum-likelihood (ML) inferred phylogenomic trees display a topology largely consistent with recent studies with either more limited taxon sampling (Zou et al. 2012; Amemiya et al. 2013) or smaller gene sampling (Broughton et al. 2013; Glasauer and Neuhauss 2014; Braasch et al. 2015) with respect to evolutionary relationships and taxonomic classification (fig. 1).

**Table 1:** List of Species Included in the Phylogenetic Analyses

Organism^source^	Scientific Name	Class	Order	Reference
*Ray-finned fish*				
Asian arowana*	*Scleropages formosus*	Actinopterygii	Osteoglossiformes	This study
European eel^Z^	*Anguilla anguilla*	Actinopterygii	Anguilliformes	Henkel et al. (2012)
Medaka^E^	*Oryzias latipes*	Actinopterygii	Beloniformes	Kasahara et al. (2007)
Blind cave fish^E^	*Astyanax mexicanus*	Actinopterygii	Characiformes	McGaugh et al. (2014)
Common carp^C^	*Cyprinus carpio*	Actinopterygii	Cypriniformes	Xu et al. (2014)
Zebrafish^E^	*Danio rerio*	Actinopterygii	Cypriniformes	Howe et al. (2013)
Amazon molly^E^	*Poecilia formosa*	Actinopterygii	Cyprinodontiformes	Unpublished
Southern platyfish^E^	*Xiphophorus maculatus*	Actinopterygii	Cyprinodontiformes	Schartl et al. (2013)
Northern pike^V^	*Esox lucius*	Actinopterygii	Esociformes	Rondeau et al. (2014)
Atlantic cod^E^	*Gadus morhua*	Actinopterygii	Gadiformes	Star et al. (2011)
Three-spined stickleback^E^	*Gasterosteus aculeatus*	Actinopterygii	Gasterosteiformes	Jones et al. (2012)
Electric eel^F^	*Electrophorus electricus*	Actinopterygii	Gymnotiformes	Gallant et al. (2014)
Spotted gar^E^	*Lepisosteus oculatus*	Actinopterygii	Lepisosteiformes	Unpublished
Nile tilapia^E^	*Oreochromis niloticus*	Actinopterygii	Perciformes	Brawand et al. (2014)
Atlantic salmon^S^^A^	*Salmo salar*	Actinopterygii	Salmoniformes	Davidson et al. (2010)
Rainbow trout^G^	*Oncorhynchus mykiss*	Actinopterygii	Salmoniformes	Berthelot et al. (2014)
Japanese puffer^E^	*Takifugu rubripes*	Actinopterygii	Tetraodontiformes	Aparicio et al. (2002)
Green spotted puffer^E^	*Tetraodon nigroviridis*	Actinopterygii	Tetraodontiformes	Jaillon et al. (2004)
*Lobe-finned fish*				
African coelacanth^E^	*Latimeria chalumnae*	Sarcopterygii	Coelacanthiformes	Amemiya et al. (2013)
^a^Lungfish^SR^	*Protopterus annectens*	Sarcopterygii	Lepidosireniformes	Amemiya et al. (2013)
*Cartilaginous fish*				
Elephant shark^A^	*Callorhinchus milii*	Chondrichthyes	Chimaeriformes	Venkatesh et al. (2014)
^b^Small-spotted catshark^SK^	*Scyliorhinus canicula*	Chondrichthyes	Carchariniformes	Wyffels et al. (2014)
^b^Little skate^SK^	*Leucoraja erinacea*	Chondrichthyes	Rajiformes	Wang et al. (2012)
*Tetrapods*				
Western clawed frog^E^	*Xenopus tropicalis*	Amphibia	Anura	Fuchs et al. (2006)
Chicken^E^	*Gallus gallus*	Aves	Galliformes	Hillier et al. (2004)
Human^E^	*Homo sapiens*	Mammalia	Primates	Venter et al. (2001)
Lizard^E^	*Anolis carolinensis*	Reptilia	Squamata	Alföldi et al. (2011)

Note.—Codes for source: A*STAR (A), CarpBase (C), Ensembl (E), efish genomics (F), Genoscope (G), SalmonDB (SA), SkateBase (SK), SRA (SR), UVic (V), ZF Genomics (Z), this study (*).

^a^Raw transcriptome reads were used.

^b^Assembled transcripts were used.

The rapid and divergent evolution of certain ray-finned fish groups is apparent in the tree from the relatively long branch lengths. Substantial evolutionary rate heterogeneity is observed within and among fish lineages by the comparison of amino acid substitutions per site calculated from branch lengths (fig. 1). Furthermore, based on Tajima’s relative rate test (supplementary material S4, Supplementary Material online), the Asian arowana was reported to have a significantly different evolutionary rate in comparison with other ray-finned fish lineages with *P* values ranging from 0 to 0.00048 (European eel). Using a Bonferroni corrected critical *P* value of 0.00098 (equivalent to α = 0.05 for a single test) results in the rejection of null hypothesis of equal rates of evolution between the arowana lineages and all other fish species.

A major difference in our estimated phylogenetic relationships to other recent studies is the placement of the arowana sample as the sister lineage to all other teleost lineages, which conflicts with morphology-based studies and more recent molecular perspectives which posit that Elopomorpha is the sister group to all other teleost lineages (Arratia 1997, 1999; Li and Wilson 1999; Diogo 2007; Broughton et al. 2013; Glasauer and Neuhauss 2014). However, our result is consistent with other studies that have the Osteoglossomorpha as the sister lineage to all other teleosts (Patterson and Rosen 1977; Lauder and Liem 1983; Nelson 1994; Inoue et al. 2003; Brinkmann et al. 2004). We look forward to more comprehensive genomic resources becoming available with greater taxon sampling for teleost fishes to allow more rigorous testing of these alternate hypotheses.

Our results support the findings of Amemiya et al. (2013) who found that the lungfish and not the coelacanth to be the closest relative to the tetrapods, which has also been a subject to much disputation (Brinkmann et al. 2004; Takezaki et al. 2004; Shan and Gras 2011). However, although we also found that the coelacanth proteins evolve at a slower rate relative to those of the tetrapods, from figure 1 it can be seen that the substitution rate in the coelacanth lineage is more than half of that for the tetrapod lineage, which is substantially faster than that observed by Amemiya et al. (2013). This discrepancy is most likely a result of the use of different protein data sets, taxon sampling, and outgroups in the two studies and provides a caveat for generalizing results from a single study even when utilizing information from a large number of genes.

### Putative Pigmentation Genes

A total of 94 different pigmentation genes were identified from our genome sequences (table 2). Only the best hit for each pigmentation gene was retained in the table and these are grouped into various functional categories related to melanophore development, components of melanosomes, melanosome construction, melanosome transport, regulation of melanogenesis, systemic effects, xanthophore development, pteridine synthesis, iridophore development, and other functions as shown by Braasch et al. (2009). This result indicates that a wide range of pigmentation genes have been retained across the teleosts and will provide a valuable resource for the study of the genetic and developmental basis for the spectacular color phenotypes of the Asian arowana.

**Table 2:** Putative Arowana Pigmentation Genes

Gene	Accession (*Homo sapiens*)	Locus ID (arowana)	PID	*e*-value	Accession (annotation)	Species
*Melanophore development*						
adam17	NP_003174.3	Z043_115716	68.98	0.00	XP_010733184.1	*Larimichthys crocea*
adamts20	NP_079279.3	Z043_106475	71.36	0.00	XP_008274326.1	*Stegastes partitus*
creb1	NP_004370.1	Z043_122987	95.37	0.00	XP_005167757.1	*Danio rerio*
ece1	NP_001106819.1	Z043_112628	80.03	0.00	CDQ77702.1	*Oncorhynchus mykiss*
Ednrb	NP_001116131.1	Z043_105076	81.50	0.00	XP_007254865.1	*Astyanax mexicanus*
Egfr	NP_958439.1	Z043_114891	—	—	—	*—*
fgfr2	NP_000132.3	Z043_104866	84.50	0.00	KKF10433.1	*La. crocea*
frem2	NP_997244.4	Z043_101382	70.22	0.00	XP_012683949.1	*Clupea harengus*
fzd4	NP_036325.2	Z043_108755	89.76	0.00	XP_012693402.1	*Cl. harengus*
gna11	NP_002058.2	Z043_106310	96.02	0.00	XP_010750457.1	*La. crocea*
gnaq	NP_002063.2	Z043_114081	86.57	0.00	XP_010735114.1	*La. crocea*
gpc3	NP_001158091.1	Z043_101235	52.03	3 × 10^−175^	XP_006639062.1	*Lepisosteus oculatus*
gpr161	NP_722561.1	Z043_116750	73.06	0.00	XP_007227875.1	*As. mexicanus*
hdac1	NP_004955.2	Z043_108210	96.71	0.00	XP_006631299.1	*Le. oculatus*
ikbkg	NP_003630.1	Z043_105761	64.16	2 × 10^−170^	XP_010903123.1	*Esox lucius*
itgb1	NP_596867.1	Z043_116749	71.96	0.00	NP_001030143.1	*D. rerio*
Kit	NP_001087241.1	Z043_118854	71.89	0.00	XP_008297546.1	*St. partitus*
lef1	NP_057353.1	Z043_100731	—	—	—	*—*
lmx1a	NP_001167540.1	Z043_108871	91.03	9 × 10^−180^	XP_008417499.1	*Poecilia reticulata*
mbtps1	NP_003782.1	Z043_104391	86.31	0.00	XP_009291810.1	*D. rerio*
mcoln3	NP_060768.8	Z043_110213	69.96	0.00	XP_006634884.1	*Le. oculatus*
mitf	NP_937801.1	Z043_105357	83.91	0.00	XP_006630679.1	*Le. oculatus*
pax3	NP_039230.1	Z043_107599	—	—	—	*—*
rab32	NP_006825.1	Z043_104281	78.47	6 × 10^−118^	XP_012671987.1	*Cl. harengus*
scarb2	NP_005497.1	Z043_105397	78.22	0.00	NP_001117983.1	*O. mykiss*
sfxn1	NP_073591.2	Z043_121119	89.10	0.00	XP_010895582.1	*E. lucius*
snai2	NP_003059.1	Z043_117231	85.88	5 × 10^−164^	XP_003759837.1	*Sarcophilus harrisii*
sox10	NP_008872.1	Z043_106242	77.78	0.00	XP_008294581.1	*St. partitus*
sox18	NP_060889.1	Z043_107469	61.33	3 × 10^−161^	XP_001337702.1	*D. rerio*
sox9	NP_000337.1	Z043_118917	79.08	0.00	XP_006635207.1	*Le. oculatus*
tfap2a	NP_001027451.1	Z043_119933	86.12	0.00	XP_006634534.1	*Le. oculatus*
trpm1	NP_001238949.1	Z043_111666	71.06	0.00	XP_006629107.1	*Le. oculatus*
trpm7	NP_060142.3	Z043_100441	82.16	0.00	XP_006628750.1	*Le. oculatus*
wnt1	NP_005421.1	Z043_120129	93.51	0.00	XP_010873444.1	*E. lucius*
wnt3a	NP_149122.1	Z043_118184	96.12	0.00	XP_008312650.1	*Cynoglossus semilaevis*
zic2	NP_009060.2	Z043_101779	88.54	0.00	XP_006638968.1	*Le. oculatus*
*Components of melanosomes*						
dct	NP_001913.2	Z043_108526	73.9	0.00	XP_008326759.1	*Cy. semilaevis*
rab32	NP_006825.1	Z043_116536	67.76	1 × 10^−88^	XP_003224067.2	*Anolis carolinensis*
rab38	NP_071732.1	Z043_122112	90.05	1 × 10^−126^	AAI50366.1	*D. rerio*
slc24a4	NP_705934.1	Z043_114251	81.84	0.00	XP_005803162.1	*Xiphophorus maculatus*
slc24a5	NP_995322.1	Z043_103396	82.06	0.00	XP_005814818.1	*X. maculatus*
tyrp1	NP_000541.1	Z043_107956	74.52	0.00	XP_005743086.1	*Pundamilia nyererei*
*Melanosome construction*						
ap3d1	NP_003929.4	Z043_120762	73.21	0.00	XP_011472829.1	*Oryzias latipes*
fig4	NP_055660.1	Z043_103115	86.55	0.00	XP_006626354.1	*Le. oculatus*
gpr143	NP_000264.2	Z043_102175	78.42	0.00	XP_012680526.1	*Cl. harengus*
hps3	NP_115759.2	Z043_100370	70.79	0.00	XP_012680760.1	*Cl. harengus*
lyst	NP_001288294.1	Z043_100757	69.99	0.00	XP_008300589.1	*St. partitus*
nsf	NP_006169.2	Z043_108447	93.61	0.00	XP_005164054.1	*D. rerio*
pldn	NP_036520.1	Z043_109414	78.42	4 × 10^−73^	XP_008274283.1	*St. partitus*
rabggta	NP_004572.3	Z043_121567	—	—	—	*—*
txndc5	NP_110437.2	Z043_116626	77.02	0.00	CDQ77189.1	*O. mykiss*
vps11	NP_068375.3	Z043_121081	90.41	0.00	XP_010863485.1	*E. lucius*
vps18	NP_065908.1	Z043_111267	85.09	0.00	XP_010892538.1	*E. lucius*
vps33a	NP_075067.2	Z043_116542	94.66	0.00	CDQ76904.1	*O. mykiss*
vps39	NP_056104.2	Z043_117047	89.05	0.00	XP_010749485.1	*La. crocea*
*Melanosome transport*						
mlph	NP_077006.1	Z043_101687	62.90	0.00	XP_005168768.1	*D. rerio*
myo5a	NP_000250.3	Z043_102448	86.24	0.00	XP_006628770.1	*Le. oculatus*
myo7a	NP_001120652.1	Z043_100931	78.91	0.00	AAI63570.1	*D. rerio*
rab27a	NP_899059.1	Z043_111973	87.89	2 × 10^−148^	XP_006628775.1	*Le. oculatus*
*Regulation of melanogenesis*						
creb1	NP_004370.1	Z043_122987	95.37	0.00	XP_005167757.1	*D. rerio*
drd2	NP_000786.1	Z043_112980	83.67	0.00	XP_006642348.1	*Le. oculatus*
mc1r	NP_002377.4	Z043_121636	76.15	4 × 10^−167^	AGC50885.1	*Cyprinus carpio*
mgrn1	NP_001135763.2	Z043_111249	85.27	0.00	XP_006637253.1	*Le. oculatus*
pomc	NP_001030333.1	Z043_103340	51.72	7 × 10^−66^	AAO17793.1	*Anguilla japonica*
*Systemic effects*						
atp6ap1	NP_001174.2	Z043_108102	66.24	0.00	XP_012682891.1	*Cl. harengus*
atp6ap2	NP_005756.2	Z043_100882	75.14	0.00	XP_012675204.1	*Cl. harengus*
atp6v0c	NP_001185498.1	Z043_125122	95.36	3 × 10^−90^	XP_008434615.1	*P. reticulata*
atp6v0d1	NP_004682.2	Z043_121933	94.48	0.00	NP_955914.1	*D. rerio*
atp6v1e1	NP_001687.1	Z043_104549	92.09	2 × 10^−143^	XP_007579195.1	*Poecilia formosa*
atp6v1f	NP_004222.2	Z043_100808	100.00	4 × 10^−81^	XP_006633325.1	*Le. oculatus*
atp6v1h	NP_998784.1	Z043_113483	90.61	0.00	XP_007260238.1	*As. mexicanus*
atp7b	NP_000044.2	Z043_122088	54.41	0.00	XP_010017200.1	*Nestor notabilis*
rps19	NP_001013.1	Z043_118939	91.67	7 × 10^−95^	XP_008329573.1	*Cy. semilaevis*
rps20	NP_001014.1	Z043_107890	100.00	4 × 10^−80^	NP_001117836.1	*O. mykiss*
*Xanthophore development*						
atp6v1e1	NP_001687.1	Z043_104549	92.09	2 × 10^−143^	XP_007579195.1	*P. formosa*
atp6v1h	NP_998784.1	Z043_113483	90.61	0.00	XP_007260238.1	*As. mexicanus*
csf1r	NP_001275634.1	Z043_118854	71.89	0.00	XP_008297546.1	*St. partitus*
ednrb	NP_001116131.1	Z043_105076	81.50	0.00	XP_007254865.1	*As. mexicanus*
ghr	NP_001229389.1	Z043_101160	57.24	0.00	BAD20706.1	*An. japonica*
pax3	NP_039230.1	Z043_107599	—	—	—	*—*
sox10	NP_008872.1	Z043_106242	77.78	0.00	XP_008294581.1	*St. partitus*
*Pteridine synthesis*						
gchi	NP_001019195.1	Z043_110449	81.94	1 × 10^−125^	XP_007231033.1	*As. mexicanus*
mycbp2	NP_055872.4	Z043_104473	91.14	0.00	XP_007251746.1	*As. mexicanus*
paics	NP_001072992.1	Z043_121868	87.94	0.00	XP_010870568.1	*E. lucius*
pcbd1	NP_000272.1	Z043_105842	95.05	1 × 10^−66^	XP_012672435.1	*Cl. harengus*
Pts	NP_000308.1	Z043_103015	81.21	2 × 10^−84^	XP_012670027.1	*Cl. harengus*
qdpr	NP_000311.2	Z043_109962	86.83	5 × 10^−129^	XP_006137052.1	*Pelodiscus sinensis*
Spr	NP_003115.1	Z043_114288	63.64	6 × 10^−126^	NP_001133746.1	*Salmo salar*
xdh	NP_000370.2	Z043_115384	69.12	0.00	XP_006636840.1	*Le. oculatus*
*Iridophore development*						
atp6v1h	NP_998784.1	Z043_113483	90.61	0.00	XP_007260238.1	*As. mexicanus*
dac	NP_001077.2	Z043_123292	73.28	0.00	ACN11084.1	*Sa. salar*
ednrb	NP_001116131.1	Z043_105076	81.50	0.00	XP_007254865.1	*As. mexicanus*
Ltk	NP_002335.2	Z043_118424	68.81	0.00	XP_010877407.1	*E. lucius*
sox10	NP_008872.1	Z043_106242	77.78	0.00	XP_008294581.1	*St. partitus*
sox9	NP_000337.1	Z043_118917	79.08	0.00	XP_006635207.1	*Le. oculatus*
trim33	NP_056990.3	Z043_115609	66.93	0.00	NP_001002871.2	*D. rerio*
vps18	NP_065908.1	Z043_111267	85.09	0.00	XP_010892538.1	*E. lucius*
vps39	NP_056104.2	Z043_117047	89.05	0.00	XP_010749485.1	*La. crocea*
*Uncategorized function*						
abhd11	NP_683711.1	Z043_117262	79.64	9 × 10^−155^	XP_010893523.1	*E. lucius*
ebna1bp2	NP_006815.2	Z043_123300	77.78	7 × 10^−146^	XP_006634973.1	*Le. oculatus*
gfpt1	NP_002047.2	Z043_101574	95.16	0.00	XP_006625541.1	*Le. oculatus*
gja5	NP_859054.1	Z043_107343	71.02	0.00	XP_008273833.1	*St. partitus*
irf4	NP_002451.2	Z043_102759	75.71	0.00	XP_006634623.1	*Le. oculatus*
kcnj13	NP_002233.2	Z043_119194	71.76	7 × 10^−173^	XP_010768290.1	*Notothenia coriiceps*
pabpc1	NP_002559.2	Z043_109572	96.20	0.00	XP_007230879.1	*As. mexicanus*
skiv2l2	NP_056175.3	Z043_112154	91.68	0.00	XP_006627067.1	*Le. oculatus*
tpcn2	NP_620714.2	Z043_115041	62.50	0.00	CDQ78014.1	*O. mykiss*

## Materials and Methods

### Sample Collection and DNA Extraction

A tail fin sample of *S. formosus* from a specimen was donated by the Malaysian Freshwater Fisheries Research Centre (FRI Glami Lemi). DNA was extracted using Qiagen Blood and Tissue DNA extraction kit (Qiagen, Hilden, Germany) according to the manufacturer’s instructions. Then, 1 µg of the purified DNA was sheared (500 bp setting) using Covaris S220 (Covaris, Woburn, MA) and prepped with Illumina TruSeq DNA Sample Preparation Kit (Illumina, San Diego, CA) according to the manufacturer’s instructions. Additionally, a 3-kb insert mate-pair library was generated using the Illumina Mate Pair Library Prep Kit. Both libraries were quantified using KAPA library quantification kit (KAPA Biosystems, Capetown, South Africa) and sequenced on the Illumina HiSeq 2000 using the 2 × 101 bp paired-end read setting (Illumina) located at the Malaysian Genomics Resource Centre.

### Genome Size Estimation based on k-mer Frequency in Sequence Reads

Genome size of *S. formosus* was approximated from k-mer frequency distributions in raw genomic reads as was done by Li et al. (2010). Frequencies of distinct 15-, 17-, 19-, and 21-mers occurring in genomic reads from the paired-end library were counted using JELLYFISH (Marçais and Kingsford 2011). The real sequencing depth (*N*) was estimated from the peak of each frequency distribution (*M*), read length (*L*), and k-mer length (*K*) correlated according to the following formula: *M* = *N* × (L − K + 1)/L. Genome size was then approximated from the division of total genomic bases by the real sequencing depth.

### Assembly and Annotation of the *Scleropages formosus* Genome

Raw reads were error corrected and preprocessed by removing low-quality reads (average Phred quality ≤20) and reads containing more than 10% ambiguous nucleotides. The resulting set of reads longer than 30 bp were assembled and scaffolded using the MSR-CA genome assembler (now renamed MaSuRCA, with default settings) (Zimin et al. 2013). Further scaffolding was carried out with reads from the mate-pair library using Scaffolder (Barton MD and Barton HA 2012). The final draft assembly consists of scaffolds longer than 200 bp. Finally, the CEGMA program (Parra et al. 2007) was used to assess the completeness of the assembly by detecting the presence of 248 highly conserved proteins within the draft genome. To compare our draft assembly with other arowana resources, transcriptomic reads generated using 454 pyrosequencing from the Asian arowana transcriptome (Shen et al. 2014) were aligned to the draft genome using GMAP (Wu and Watanabe 2005). Unmapped transcriptomic reads were further characterized by a BLASTN (Altschul et al. 1990) search against the NT database on NCBI.

Arowana transcriptome reads were downloaded (SRA: SRR941557, SRR941783, SRR941785), preprocessed with QTrim (default settings) (Shrestha et al. 2014), and assembled de novo using IDBA-tran (–max_isoforms 10 –maxk 80) (Peng et al. 2013). To predict protein-coding genes, MAKER (Cantarel et al. 2008) was run on the arowana genome using the assembled arowana transcriptome and Ensembl proteins from zebrafish (*Danio rerio*), Nile tilapia (*Oreochromis niloticus*), medaka (*Oryzias latipes*), and Japanese puffer (*Takifugu rubripes*) as evidence. Repetitive regions were masked with all organisms in RepBase. MAKER was run iteratively to train the SNAP (Korf 2004) gene predictor in a bootstrap fashion to improve the predictor’s performance, and final MAKER predictions were made using the trained SNAP as well as Augustus trained with the zebrafish species model. Functional annotation of the predicted sequences was performed with a BLASTP (Altschul et al. 1990) search (*e*-value threshold of 1 × 10^−10^) against vertebrate proteins in NCBI’s NR database. A 70% blast hit coverage cut-off (based on subject length) was also applied to obtain confident annotations. Unannotated protein sequences were then searched against all sequences in NCBI’s NR database with the same *e*-value and hit coverage cut-offs. Gene ontologies, protein domains, and families were identified with InterProScan (Jones et al. 2014). tRNA genes in the assembly were detected by MAKER using tRNAscan (Lowe and Eddy 1997), while RNAmmer (Lagesen et al. 2007) was used to predict rRNA sequences.

### Orthology Inference

Data selection for phylogenomic analyses is controversial and centers on issues of data quality and quantity and on benefits of taxon sampling versus high data coverage that minimizes alignment gaps (Laurin-Lemay et al. 2012; Amemiya et al. 2013; Betancur-R et al. 2013; Misof et al. 2013; Salichos and Rokas 2013). We take a conservative approach that minimizes gaps in the supermatrix and use several ways to carefully distinguish orthologs from paralogs to assemble a high quality phylogenomic data set, ensuring the estimation of a robust and accurate tree, including the placement of the deeper lineages in the tree.

First, because conserved genes make for the best phylogenomic markers (Betancur-R et al. 2013), Hidden Markov Model (HMM) profiles from the TreeFam database (Schreiber et al. 2014) of gene families conserved across 104 other animal species were used to identify these conserved protein sequences in the arowana genome. For all species, protein sequences longer than 100 amino acids were scanned for sequence homology to gene families in the TreeFam database (version 9) (Schreiber et al. 2014) using *hmmsearch* (Eddy 2011) (*e*-value threshold of 1 × 10^−10^) and gene families having sequence homology to at least one protein in all 27 species were retained for subsequent orthology inference. Orthology inference from these protein clusters was conducted with scripts from the pipeline recently described by Yang and Smith (2014), which employs a tree-based approach to first identify paralogs, prune spurious branches, and finally identify orthologs. Briefly, protein sequences in each gene family were aligned and trimmed with the *fasta_to_tree.py* script. In addition, clusters containing paralogs were limited during orthology inference by implementing a tree-based approach on individual sequence clusters, along with additional pruning steps, to separate paralogs and orthologs (Yang and Smith 2014). Due to computational limitations, we modified the pipeline to use IQ-TREE (Nguyen et al. 2015) to build smaller gene trees (less than 1,000 sequences) and FastTreeMP (Price et al. 2010) for larger gene trees. For each tree, tips longer than 0.5 (=absolute tip cut-off) or longer than 0.2 and ten times longer than its nearby tips (=relative tip cut-off) were trimmed with *trim_tips.py*. Monophyletic tips belonging to the same taxon were masked with *mask_tips_by_taxonID_genomes.py*. Internal branches longer than 0.3, which may be separating orthologous groups, were cut with *cut_long_internal_branches.py* and only trees containing sequences from all 27 species were retained, thus reducing the amount of missing data and lowering the potential for nonphylogenetic signals (Borowiec et al. 2015). Protein sequence alignment, alignment trimming, and gene tree building were repeated for remaining sequences for each tree. Orthology inference was then carried out on the newly inferred trees with paralogy pruning by maximum inclusion using the *prune_paralogy_MI.py* script (relative tip cut-off 0.2, absolute tip cut-off 0.5, minimum taxa 27), which iteratively extracts the subtree containing the most taxa without taxon duplication. Protein sequences in each cluster were aligned with *mafft_wrapper.py*, each alignment was trimmed with *pep_gblocks_wrapper.py*, and all alignments were finally concatenated into a supermatrix.

Orthology calls in teleosts, and specifically for Osteoglossomorphs and Elopomorphs, are not as simple and are complicated by divergent evolution in genes as a result of multiple rounds of genome duplication prior to teleost diversification (Braasch et al. 2015). Although we have taken several strict measures to identify orthologs and exclude paralogs, it is important to note that it is extremely challenging to ensure that all identified protein sequences in each cluster are truly orthologous.

### Phylogenetic Analysis

Phylogenetic analysis was done based on amino acid alignments for a total of 27 species (table 1). For organisms lacking available proteome data sets, namely the lungfish, little skate, and small-spotted catshark, protein sequences were obtained from their respective transcriptomes. For the lungfish specifically, raw Illumina RNA-seq reads (SRA: SRR505721–SRR505726) were assembled with the Trinity assembler (Grabherr et al. 2011). All transcriptomes were translated with Transdecoder (http://transdecoder.sourceforge.net/, last accessed April 14, 2015).

Each ortholog is treated as a separate data block and used as input to PartitionFinder (branchlengths = linked, model_selection = AICc, search = rcluster) (Lanfear et al. 2014) to estimate the best-fit partitioning schemes and models of protein evolution. Based on these results, ML analysis was conducted with RAxML (Stamatakis 2014) under the recommended partitions and substitution models. A total of 100 trees were generated using distinct random seeds and the tree with the best likelihood value was chosen as the final tree topology. Nodal support was represented by bootstrap replicates with the *autoMRE* convergence criterion (Pattengale et al. 2009). A Bayesian inference using the same supermatrix partitioned into each ortholog was also carried out using ExaBayes (Aberer et al. 2014). Four independent chains were run for 2 million generations and sampled every 500 generations. With 25% of initial samples discarded as burn-in, runs were considered to have converged when the average standard deviation of split frequencies is less than 1%. Both ML and BI phylogenetic trees were rooted using the Chondrichthyes as the outgroup and visualized with MEGA6 (Tamura et al. 2013).

### Rate of Molecular Evolution

To compare evolutionary rates of the Asian arowana versus other ray-finned fish lineages, the rate of molecular evolution for each fish lineage was calculated by adding branch lengths from the end of each terminal branch to the node where the split between ray-finned fish and lobe-finned fish (and tetrapods) occurred (fig. 1, orange star). In addition, the Tajima’s relative rate test (Tajima 1993) was implemented, as done by Amemiya et al. (2013) to test for equal rates between lineages. Using MEGA6 (Tamura et al. 2013), Tajima’s relative rate tests (with missing positions and gaps eliminated) were conducted for comparisons between the Asian arowana and other ray-finned fishes, with a member of the Chondricthyes set as outgroup.

### Identification of Putative Pigmentation Genes

Predicted protein sequences for arowana were screened for putative pigmentation genes using a list curated by Braasch et al. (2009). Using their homologs in humans (table 2), arowana proteins were searched against pigment genes using BLASTP (Altschul et al. 1990) with an *e*-value threshold of 1 × 10^−40^ and subsequently filtered with a hit coverage cut-off of 70%. The best hit for each pigment gene was chosen as a candidate to test for the presence of conserved domains by using the Batch CD-Search tool (Marchler-Bauer and Bryant 2004) to search against the Conserved Domain Database (Marchler-Bauer et al. 2014).

## Supplementary Material

Supplementary materials S1–S4 are available at *Genome Biology and Evolution* online (http://www.gbe.oxfordjournals.org/).

Supplementary Data
