# Supplementary material for: Whole Genome Sequencing of the Asian Arowana (Scleropages formosus) Provides Insights into the Evolution of Ray-Finned Fishes
Source: Genome Biol Evol. 2015 Oct 6;7(10):2885–95. doi: 10.1093/gbe/evv186 (PMC4684697; doi:10.1093/gbe/evv186)
Supplement: Supplementary Data [file supp_7_10_2885__index.html]

Whole genome sequencing of the Asian arowana (Scleropages formosus) provides insights into the evolution of ray-finned fishes. — Whole Genome Sequencing of the Asian Arowana (Scleropages formosus) Provides Insights into the Evolution of Ray-Finned Fishes — Supplementary Data 

# Whole Genome Sequencing of the Asian Arowana (*Scleropages formosus*) Provides Insights into the Evolution of Ray-Finned Fishes

## Supplementary Data

files

- Supplementary Data - txt file
- Supplementary Data - phy file
- Supplementary Data - txt file
- Supplementary Data - xlsx file
